# Supplementary material for: Comparative Analysis of Chemical Profile and Biological Activity of Juniperus communis L. Berry Extracts
Source: Plants (Basel). 2023 Sep 27;12(19):3401. doi: 10.3390/plants12193401 (PMC10574284; doi:10.3390/plants12193401)
Supplement: Supplementary file 1 [file plants-12-03401-s001.zip › plants-2579648-supplementary.pdf]

Table S1. Influence of factors on extraction yield according to ANOVA multiple analysis of variance (p <0.05 is considered significant).

Factor	P-*m*	P-*usm*	C-*m*	C-*usm*	A-*m*	A-*usm*	M-*m*	M-*usm*	E-*m*	E- *usm*
P-*m*[1]		0.000	0.305	0.000	0.000	0.000	0.000	0.000	0.000	0.000
P-*usm*			0.000	0,116	0.000	0.000	0.000	0.000	0.000	0.000
C-*m*	0.305			0.000	0.000	0.000	0.000	0.000	0.000	0.000
C-*usm*		0.116			0.000	0.000	0.000	0.000	0.000	0.000
A-*m*						0.000	0.000	0.000	0.000	0.000
A-*usm*							0.073	0.000	0.000	0.000
M-*m*						0.073		0.000	0.000	0.000
M-*usm*									0.000	0.000
E-*m*										0.000
E- *usm*										

[1]P-*m* – Pentane, maceration; P-*usm* – Pentane, ultrasound-assisted maceration; C-*m* – Chloroform, maceration; C-*usm* – Chloroform, ultrasound-assisted maceration; A-*m* – Acetone, maceration; A-*usm* – Acetone, ultrasound-assisted maceration; M-*m* – Methanol, maceration; M- *usm* – Methanol, ultrasound-assisted maceration; E-*m* – 70% ethanol, maceration; E-*usm* – 70% ethanol, ultrasound-assisted maceration.

Table S2. Influence of factors on total phenolic compounds according to ANOVA multiple analysis of variance (p <0.05 is considered significant).

Factor	P-*m*	P-*usm*	C-*m*	C-*usm*	A-*m*	A-*usm*	M-*m*	M-*usm*	E-*m*	E- *usm*
P-*m*[1]		1.000	0.000	0.000	0.000	0.000	0.000	0.000	0.000	0.000
P-*usm*	1.000		0.000	0.000	0.000	0.000	0.000	0.000	0.000	0.000
C-*m*				0,006	0.000	0.000	0.000	0.000	0.000	0.000
C-*usm*			0.006		0.000	0.000	0.000	0.000	0.000	0.000
A-*m*						0.000	0.998	0.000	0.000	0.000
A-*usm*							0.000	0.000	0.000	0.000
M-*m*					0.998			0.000	0.000	0.000
M-*usm*									0.000	0.000
E-*m*										0.000
E- *usm*										

[1]P-*m* – Pentane, maceration; P-*usm* – Pentane, ultrasound-assisted maceration; C-*m* – Chloroform, maceration; C-*usm* – Chloroform, ultrasound-assisted maceration; A-*m* – Acetone, maceration; A-*usm* – Acetone, ultrasound-assisted maceration; M-*m* – Methanol, maceration; M- *usm* – Methanol, ultrasound-assisted maceration; E-*m* – 70% ethanol, maceration; E-*usm* – 70% ethanol, ultrasound-assisted maceration.

Table S3. Influence of factors on total sugars according to ANOVA multiple analysis of variance (p <0.05 is considered significant).

Factor	P-*m*	P-*usm*	C-*m*	C-*usm*	A-*m*	A-*usm*	M-*m*	M-*usm*	E-*m*	E- *usm*
P-*m*[1]		0.000	0.000	0.000	0.000	0.000	0.000	0.000	0.000	0.000
P-*usm*			0.000	0.000	0.000	0.000	0.000	0.000	0.000	0.000
C-*m*				0.977	0.000	0.000	0.000	0.000	0.000	0.000
C-*usm*			0.977		0.000	0.000	0.000	0.000	0.000	0.000
A-*m*						0.000	0.000	1.000	0.000	0.000
A-*usm*							0.000	0.000	0.000	0.000

M-*m*								0.000	0.000	0.000
M-*usm*					1.000				0.000	0.000
E-*m*										0.000
E- *usm*										

[1]P-*m* – Pentane, maceration; P-*usm* – Pentane, ultrasound-assisted maceration; C-*m* – Chloroform, maceration; C-*usm* – Chloroform, ultrasound-assisted maceration; A-*m* – Acetone, maceration; A-*usm* – Acetone, ultrasound-assisted maceration; M-*m* – Methanol, maceration; M- *usm* – Methanol, ultrasound-assisted maceration; E-*m* – 70% ethanol, maceration; E-*usm* – 70% ethanol, ultrasound-assisted maceration.

Table S4. Influence of factors on total flavonoids according to ANOVA multiple analysis of variance (p <0.05 is considered significant).

**Factor**	**P-*m***	**P-*usm***	**C-*m***	**C-*usm***	**A-*m***	**A-*usm***	**M-*m***	**M-*usm***	**E-*m***	**E- *usm***
P-*m*[1]		0,631	0.000	0.000	0.000	0.000	0.000	0.000	0.000	0.000
P-*usm*	0.631		0.000	0.000	0.000	0.000	0.000	0.000	0.000	0.000
C-*m*				0.000	0.000	0.000	0.000	0.000	0.000	0.000
C-*usm*					0.000	0.000	0.000	0.000	0.000	0.000
A-*m*						0,000	0.993	0.000	0.000	0.000
A-*usm*							0.000	0.000	0.000	0.000
M-*m*					0.993			0.000	0.000	0.000
M-*usm*									0.000	0.000
E-*m*										0.000
E- *usm*										

[1]P-*m* – Pentane, maceration; P-*usm* – Pentane, ultrasound-assisted maceration; C-*m* – Chloroform, maceration; C-*usm* – Chloroform, ultrasound-assisted maceration; A-*m* – Acetone, maceration; A-*usm* – Acetone, ultrasound-assisted maceration; M-*m* – Methanol, maceration; M- *usm* – Methanol, ultrasound-assisted maceration; E-*m* – 70% ethanol, maceration; E-*usm* – 70% ethanol, ultrasound-assisted maceration.

Table S5. Influence of factors on total terpenoids according to ANOVA multiple analysis of variance (p <0.05 is considered significant).

**Factor**	**P-*m***	**P-*usm***	**C-*m***	**C-*usm***	**A-*m***	**A-*usm***	**M-*m***	**M-*usm***	**E-*m***	**E- *usm***
P-*m*[1]		0.256	0.000	0.995	0.000	0.000	0.000	0.000	0.000	0.000
P-*usm*	0.256		0.000	0.028	0.000	0.000	0.000	0.000	0.000	0.000
C-*m*				0.007	0.000	0.000	0.000	0.000	0.000	0.000
C-*usm*	0.995	0.028	0.007		0.000	0.000	0.000	0.000	0.000	0.000
A-*m*						0.000	0.000	0.000	0.000	0.000
A-*usm*							0.998	0.000	0.000	0.000
M-*m*						0.998		0.000	0.000	0.000
M-*usm*									0.000	0.000
E-*m*										0.989
E- *usm*									0.989	

[1]P-*m* – Pentane, maceration; P-*usm* – Pentane, ultrasound-assisted maceration; C-*m* – Chloroform, maceration; C-*usm* – Chloroform, ultrasound-assisted maceration; A-*m* – Acetone, maceration; A-*usm* – Acetone, ultrasound-assisted maceration; M-*m* – Methanol, maceration; M- *usm* – Methanol, ultrasound-assisted maceration; E-*m* – 70% ethanol, maceration; E-*usm* – 70% ethanol, ultrasound-assisted maceration.

Table S6. Influence of factors on content of ascorbic acid according to ANOVA multiple analysis of variance ($p < 0.05$ is considered significant).

Factor	P-*m*	P-*usm*	C-*m*	C-*usm*	A-*m*	A-*usm*	M-*m*	M-*usm*	E-*m*	E- *usm*
P-*m*[1]		0.000	0.000	0.000	0.000	0.000	0.000	0.000	0.000	0.000
P-*usm*			0.000	0.000	0.000	0.000	0.000	0.000	0.000	0.000
C-*m*				0.000	0.000	0.000	0.000	0.000	0.000	0.000
C-*usm*					0.000	0.000	0.000	0.000	0.000	0.000
A-*m*						0.000	0.002	0.000	0.000	0.000
A-*usm*							0.000	0.000	0.000	0.000
M-*m*					0.002			0.328	0.000	0.000
M-*usm*							0.328		0.000	0.000
E-*m*										0.000
E- *usm*										

[1]P-*m* – Pentane, maceration; P-*usm* – Pentane, ultrasound-assisted maceration; C-*m* – Chloroform, maceration; C-*usm* – Chloroform, ultrasound-assisted maceration; A-*m* – Acetone, maceration; A-*usm* – Acetone, ultrasound-assisted maceration; M-*m* – Methanol, maceration; M- *usm* – Methanol, ultrasound-assisted maceration; E-*m* – 70% ethanol, maceration; E-*usm* – 70% ethanol, ultrasound-assisted maceration.

Table S7. GC-MS identification and quantification of components of 1 and 2 year old J. communis L. berries and the ratio of the mass of an ion to its charge.

№[2]	t_R[3], (min)	Component	**m/z and peak relative intensity (%)	[4]ω, %				Class of compounds
				[1]JB1E *	JB1A *	JB2E *	JB2A *	
1.	3.962	1-Hydroxybut-3-en-2-one	86 ([M+]***, 3 %), 55 (100%), 27 (%), 31 (%)	nd[5]	nd	1.913 ±0.005	nd	ketone
2.	4.063	Acetol	74 ([M+], 7 %), 43 (100%), 31 (23%), 15 (18%)	nd	nd	3.439 ±0.009	0.828 ±0.006	α-hydroxy ketones
3.	4.199	2-Hydroxy-3-oxobutanal	102 ([M+], 7 %), 43 (100%), 42 (8%),	nd	nd	2.427 ±0.007	nd	beta-ketoaldehydes
4.	4.675	Glyceraldehyde	90 ([M+], 0 %), 31 (100%), 29 (71%), 43 (52%)	nd	nd	0.785 ±0.004	3.548 ±0.009	Monosaccharide
5.	5.288	Methyl 2-oxopropanoate	102 ([M+], 11 %), 43 (100%), 42 (8%), 15 (6%)	nd	nd	1.131 ±0.006	nd	Keto acid
6.	5.507	Dihydroxyacetone	90 ([M+], 0 %), 31 (100%), 43 (33%), 29 (26%)	0.625 ±0.005	1.414 ±0.003	39.830 ±0.009	17.487 ±0.009	Monosaccharide
7.	6.016	2-Cyclopenten-1-one, 2-hydroxy-	98 ([M+], 100 %), 55 (63%), 42 (55%), 41 (34%)	nd	nd	0.642 ±0.003	nd	Lactone
8.	6.310	α-Pinene	136 ([M+], 1 %), 93 (100%), 91 (41%), 92 (38%)	42.272 ±0.011	40.944 ±0.013	20.558 ±0.008	40.388 ±0.014	Monoterpene
9.	6.783	1,2,3-Propanetriol	92 ([M+], 0 %), 61 (100%), 43 (78%), 31 (39%)	nd	nd	1.868 ±0.004	7.337 ±0.008	Polyalcohol
10.	6.883	Sabinene	136 ([M+], 15 %), 93 (100%), 91 (42%), 77 (38%)	0.564 ±0.008	0.482 ±0.004	nd	nd	Bicyclic monoterpene
11.	6.944	2-Hydroxy-γ-butyrolactone	102 ([M+], 0 %), 57 (100%), 58 (%), 29 (%)	nd	nd	1.279 ±0.011	nd	Lactone
12.	7.033	β-Myrcene	136 ([M+], 1 %), 93 (100%), 41 (98%), 69 (85%)	8.467 ±0.011	7.491 ±0.009	3.553 ±0.005	6.411 ±0.007	Monoterpene
13.	7.456	δ-3-Carene	136 ([M+], 30 %), 93 (100%), 91 (50%), 92 (31%)	1.155 ±0.002	1.063 ±0.005	nd	nd	Bicyclic monoterpene
14.	7.757	Limonene	136 ([M+], 23 %), 68 (100%), 93 (50%), 67 (44%)	0.546 ±0.006	0.502 ±0.004	nd	nd	Cyclic monoterpene
15.	8.284	4-Methyl-1H-pyrazole-3-carboxylic acid	126 ([M+], 100 %), 55 (100%), 83 (22%), 70 (10%)	nd	nd	0.829 ±0.007	nd	Pyrazole

16.	9.574	Verbenol	152 ([M+], 0 %), 91 (100%), 41 (75%), 94 (72%)	nd	nd	0.964 ±0.005	0.852 ±0.006	Monoterpene alcohol
17.	10.341	2H-Pyran-2-methanol, tetrahydro-	116 ([M+], 0 %), 85 (100%), 29 (58%), 41 (57%)	nd	nd	0.911 ±0.008	nd	Pyran
18.	10.517	Verbenone	150 ([M+], 45 %), 107 (100%), 135 (69%), 39 (64%)	nd	nd	0.324 ±0.002	0.865 ±0.004	Monoterpene ketone
19.	10.782	1,2,3-Propanetriol, 1,2-diacetate	176 ([M+], 0 %), 43 (100%), 103 (18%), 44 (6%)	nd	nd	0.772 ±0.003	nd	Lipid (diacylglycerol)
20.	11.033	Methyl citronellate	184 ([M+], 4 %), 69 (100%), 95 (76%), 110 (75%)	0.428 ±0.006	0.470 ±0.004	nd	nd	Acyclic monoterpenoid
21.	11.581	Bornyl acetate	196 ([M+], 3 %), 95 (100%), 43 (76 %), 93 (46%)	nd	0.438 ±0.002	nd	nd	Bicyclic monoterpenoid
22.	13.061	β-Elemene	204 ([M+], 2 %), 93 (100%), 81 (88%), 107 (65%)	0.607 ±0.003	0.393 ±0.004	0.534 ±0.003	nd	Sesquiterpene
23.	13.552	Caryophyllene	204 ([M+], 7 %), 41 (100%), 93 (79%), 69 (76%)	2.427 ±0.011	2.241 ±0.009	1.053 ±0.007	1.081 ±0.008	Sesquiterpene
24.	13.717	(E)-β-Farnesene	204 ([M+], 4 %), 41 (100%), 69 (79%), 93 (45%)	0.375 ±0.003	0.347 ±0.004	nd	nd	Sesquiterpene
25.	14.007	α-Humulene (α-Caryophyllene)	204 ([M+], 5 %), 93 (100%), 80 (39%), 121 (25%)	1.720 ±0.011	1.537 ±0.012	0.546 ±0.009	0.935 ±0.008	Sesquiterpene
26.	14.312	Germacrene D	204 ([M+], 17 %), 161 (100%), 105 (85%), 91 (58%),	14.440 ±0.010	14.769 ±0.013	4.132 ±0.007	6.823 ±0.003	Sesquiterpene
27.	14.487	Bicyclogermacrene	204 ([M+], 16 %), 121 (100%), 93 (68%), 41 (56%)	0.493 ±0.003	nd	nd	nd	Sesquiterpene
28.	15.444	Germacrene D-4-ol	222 ([M+], 2 %), 81 (100%), 43 (77%), 41 (40%)	1.766 ±0.008	1.941 ±0.009	0.857 ±0.007	1.220 ±0.005	Sesquiterpene
29.	17.153	Myo-inositol	194 ([M+], 0 %), 87 (100%), 73 (90%), 85 (61%)	19.172 ±0.014	12.673 ±0.009	11.265 ±0.011	4.766 ±0.007	Polysaccharide
30.	21.504	Epimetendiol	304 ([M+], 1 %), 105 (100%), 91 (93%), 93 (88%)	1.891 ±0.005	3.654 ±0.007	nd	nd	Steroid
31.	21.514	1,3,6,10-Cyclotetradecatetraene, 3,7,11-trimethyl-14-(1-methylethyl)-, [S-(E,Z,E,E)]-	272 ([M+], 9 %), 93 (100%), 105 (80%), 107 (76%)	nd	nd	nd	1.786 ±0.004	Cembrane diterpenoids
32.	22.908	Isocembrol	290 ([M+], 1 %), 43 (100%), 81 (58 %), 41 (54 %)	nd	nd	0.388 ±0.008	1.221 ±0.006	Diterpenoid
33.	22.923	(1R,4aR,5S)-5-((E)-5-Methoxy-3-methylpent-3-en-1-yl)-1,4a-dimethyl-6-methylenedecahydronaphthalene-1-carbaldehyde	318 ([M+], 1 %), 81 (100%), 107 (80%), 55 (76%)	0.754 ±0.007	1.830 ±0.008	nd	nd	Aromatic aldehyde
34.	23.159	Dehydroabietinol	286 ([M+], 38 %), 253 (100%), 271 (93%), 173 (53%)	nd	0.721 ±0.004	nd	nd	Diterpenoid
35.	23.783	Cyclopropaneoctanoic acid, 2-[[2-[(2-ethylcyclopropyl)methyl]cyclopropyl]methyl]-, methyl ester	334 ([M+], 1 %), 41 (100%), 55 (93%), 67 (89%)	0.948 ±0.003	2.525 ±0.008	nd	nd	Fatty acid ester
36.	23.933	(1R,4aR,5S)-5-[(E)-5-Hydroxy-3-methylpent-3-enyl]-1,4a-dimethyl-6-methylidene-3,4,5,7,8,8a-hexahydro-2H-naphthalene-1-carbaldehyde	304 ([M+], 1 %), 81 (100%), 107 (89%), 123 (78%)	0.775 ±0.005	1.899 ±0.011	nd	nd	Cyclic aldehyde
37.	25.725	Methyl 5,11,14-eicosatrienoate	320 ([M+], 2 %), 67 (100%), 81 (79%), 79 (71%)	0.575 ±0.004	nd	nd	nd	Fatty acid esters
38.	34.863	Nonacosan-10-ol	424 ([M+], 0 %), 83 (100%), 97 (66%), 57 (59%)	nd	2.666 ±0.012	nd	4.452 ±0.015	Fatty alcohol

[1]JB1E – Juniper berries in the first year of maturation, 70% ethanol; JB1A – Juniper berries in the first year of maturation, acetone; JB2E – Juniper berries in the second year of maturation, 70% ethanol; JB2A – Juniper berries in the second year of maturation, acetone; [2]Peak number; [3] t_R – retention time; [4]ω - mass fraction of the component as area in % of the 100.00% of all identified peaks; [5]nd: not detected; * - components with content ⩾ 0.040 % wt. of extract are given, each value represents mean ± SD (n = 3, p = 0.95); ** m/z - the ratio of the mass of an ion to its charge; *** - [M+] (EI - electron ionization at 70 eV)

Figure S1: GC-MS chromatograms of samples. (A) berries of 1st year, acetone *; (B)* berries of 2nd year, acetone*; (C)* berries of 1st year, 70% ethanol *; (D)* berries of 2nd year, 70% ethanol.

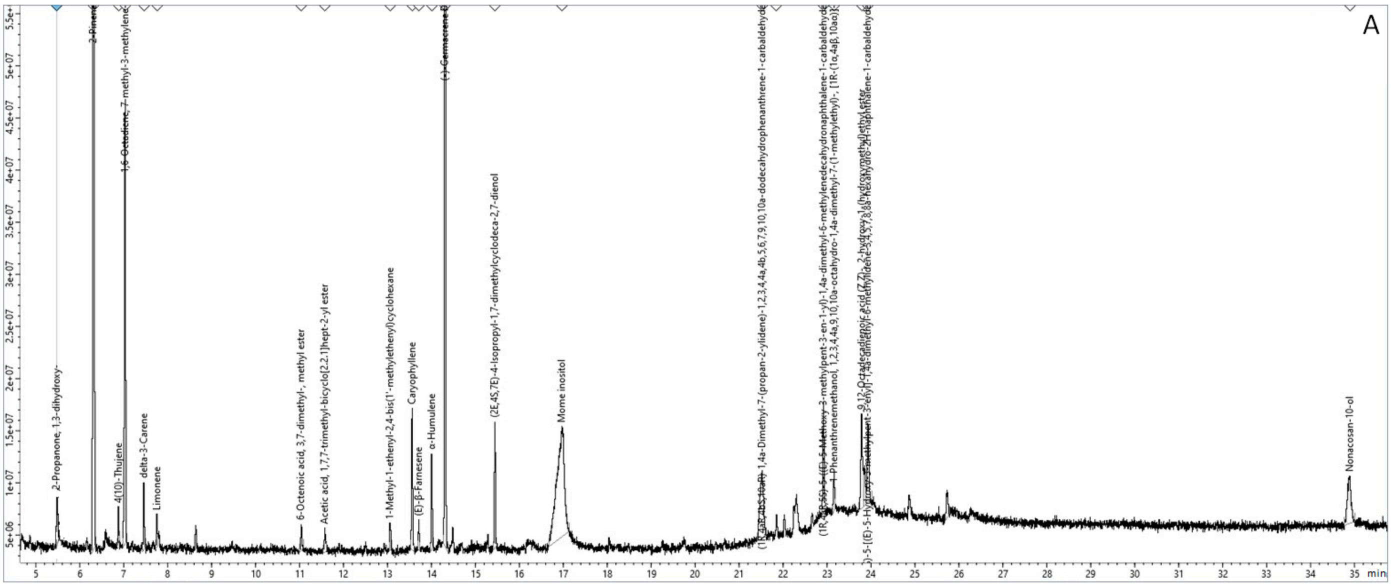

(A)

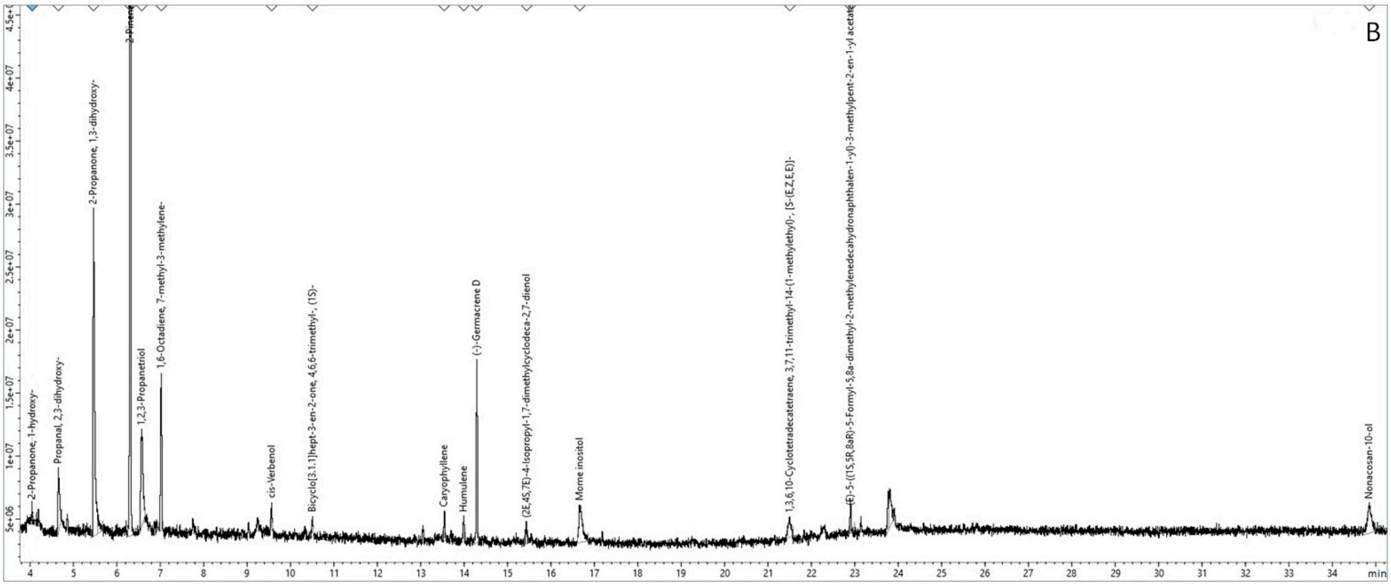

(B)

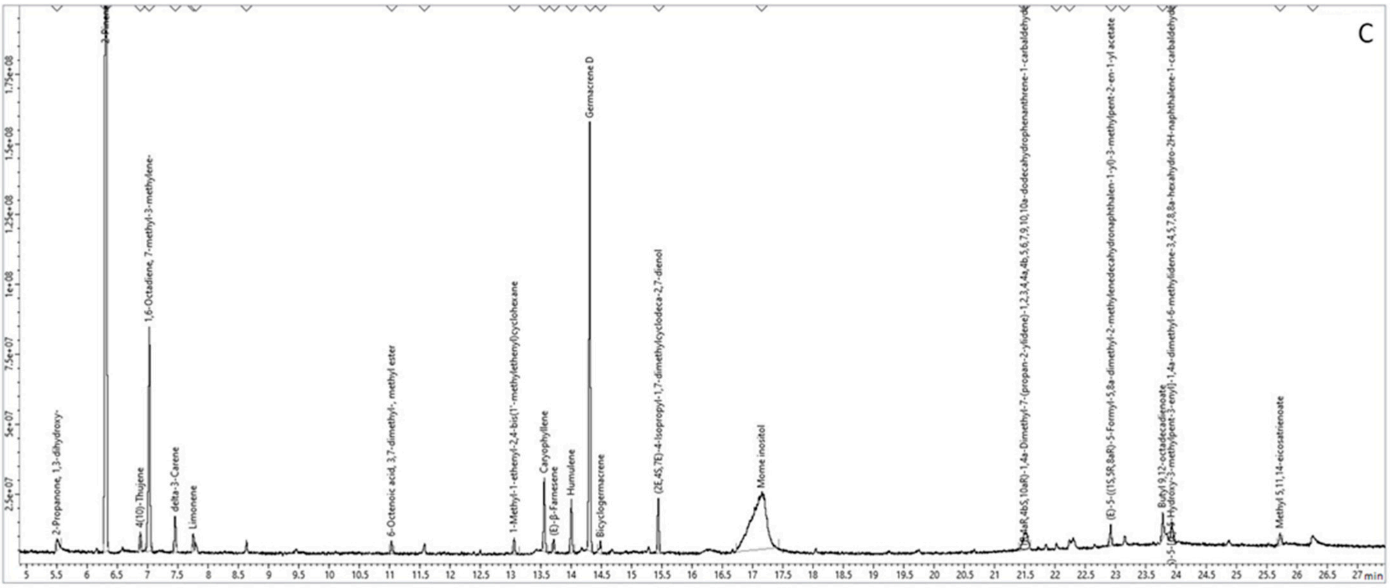
C
2-Propanone, 1,3-dihydroxy-
2-Pinene
4(10)-Thujene
1,6-Octadiene, 7-methyl-3-methylene-
delta-3-Carene
Limonene
6-Octenoic acid, 3,7-dimethyl-, methyl ester
1-Methyl-1-ethenyl-2,4-bis(1'-methylethenyl)cyclohexane
Caryophyllene
(E)-β-Farnesene
Humulene
Germacrene D
Bicyclogermacrene
(2E,4S,7E)-4-Isopropyl-1,7-dimethylcyclodeca-2,7-dienol
Mome inositol
(E)-5-((1S,5R,8aR)-5-Formyl-5,8a-dimethyl-2-methylenedecahydronaphthalen-1-yl)-3-methylpent-2-en-1-yl acetate
Butyl 9,12-octadecadienoate
Methyl 5,11,14-eicosatrienoate
min

(C)

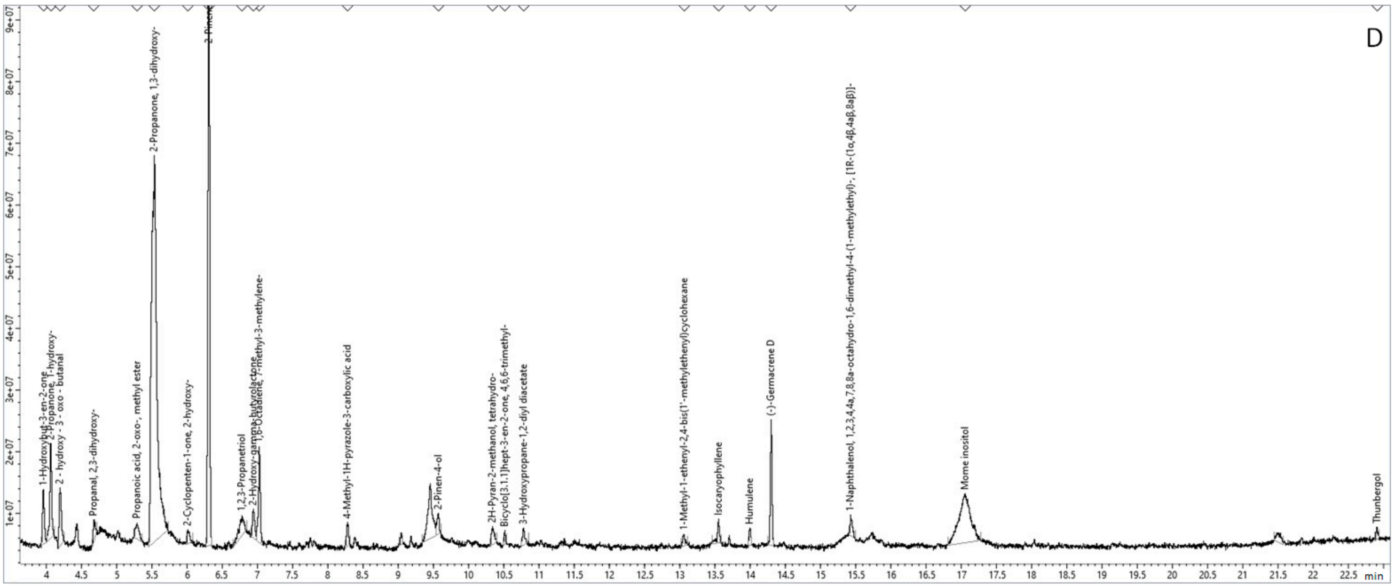
D
2-Propanone, 1-hydroxy-
2 - hydroxy - 3 - oxo - butanal
Propanal, 2,3-dihydroxy-
Propanoic acid, 2-oxo-, methyl ester
2-Propanone, 1,3-dihydroxy-
2-Cyclopenten-1-one, 2-hydroxy-
2-Pinene
1,2,3-Propanetriol
1,6-Octadiene, 7-methyl-3-methylene-
4-Methyl-1H-pyrazole-3-carboxylic acid
2-Pinen-4-ol
2H-Pyran-2-methanol, tetrahydro-
Bicyclo[3.1.1]hept-3-en-2-one, 4,6,6-trimethyl-
3-Hydroxypropane-1,2-diyl diacetate
1-Methyl-1-ethenyl-2,4-bis(1'-methylethenyl)cyclohexane
Isocaryophyllene
Humulene
(-)-Germacrene D
1-Naphthalenol, 1,2,3,4,4a,7,8,8a-octahydro-1,6-dimethyl-4-(1-methylethyl)-, [1R-(1α,4β,4aβ,8aβ)]-
Mome inositol
Thunbergol
min

(D)
